# Molecular Characterization of Nascent and Aged Sea Spray Aerosol

**DOI:** 10.1021/acsearthspacechem.4c00412

**Published:** 2025-05-19

**Authors:** Dilini K. Gamage, Elias Hasenecz, Glorianne P. Dorcé, Kathryn J. Mayer, Jon S. Sauer, Christopher Lee, Kimberly A. Prather, Elizabeth A. Stone

**Affiliations:** † Department of Chemistry, 4083 University of Iowa, Iowa City, Iowa 52242, United States; ‡ Department of Chemistry and Biochemistry, 8784 University of California, La Jolla, San Diego, California 92093, United States; § 70015 Scripps Institution of Oceanography, University of California, La Jolla, San Diego, California 92037, United States

**Keywords:** organic carbon, sea spray aerosol, organosulfates, fatty acids, alkyl amines, chemical aging, secondary organic aerosol, marine aerosol

## Abstract

The chemical aging of sea spray aerosol (SSA) was examined in the Sea Spray Chemistry and Particle Evolution (SeaSCAPE) experiment in which nascent SSA particles were generated from seawater by breaking waves in a glass wave channel. Particles and gases in the air in the wave channel headspace were aged in an oxidative flow reactor. Nascent SSA (before reaction) and aged SSA (after reaction) particles were chemically analyzed for inorganic ions, organic carbon (OC), and select organic species, including organosulfates, fatty acids, and alkyl amines. Nascent SSA mass primarily consisted of inorganic ions associated with sea salt. On average, OC accounted for 52% of particle mass <0.25 μm and 0.2% of mass in both supermicron and submicron particles, with an increase in OC relative to Na^+^ with decreasing particle size. The aging process increased the sulfate, phosphate, nitrate, ammonium, and OC concentrations relative to sodium. The largest increases in the sulfate and OC to Na^+^ ratios (by factors of 7 and 5) in aged SSA were observed in particles with diameters <0.25 μm. Organosulfates, which accounted for approximately 1% of SSA OC mass in PM_1.0_, were enhanced in aged SSA and indicated the formation of low-volatility secondary organic aerosol products associated with aging biological molecules such as unsaturated fatty acids, isoprene, and monoterpenes. For example, isoprene-derived organosulfates (e.g., 2-methyltetrol sulfate, C_5_H_11_SO_7_^–^; *m*/*z* 215.0225) increased by a factor of 40 in samples of aged SSA and marine volatile organic compounds. Among the strongest organosulfate signals in nascent and aged SSA were alkyl organosulfates, which are known to be anthropogenic surfactants in coastal waters. Homologous series of saturated and unsaturated fatty acids, fatty acid derivatives, and alkyl amines were also identified in nascent and aged SSA, with some species enhanced by aging (i.e., diethylamine) and others not. Together, these bulk and molecular analyses provide insight into molecular modifications that occur upon the chemical aging of nascent SSA.

## Graphical abstract

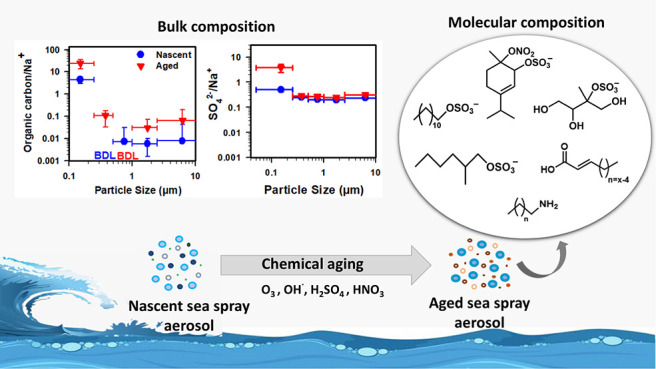

## 1 Introduction

The ocean represents a major source of atmospheric aerosols by mass^1^ in the form of sea spray aerosol (SSA) produced by breaking waves at the ocean surface. SSA affects global radiation and climate by scattering solar radiation and by acting as cloud condensation nuclei and ice nuclei.^2–4^ In SSA, the ratio of organic matter to sea salt is higher than in seawater, especially in submicron particles, leading to significant organic-to-salt enrichment^2,5–8^ on the order of 10^3^ to 10^5^.^9,10^ The composition of inorganic and organic contents in SSA varies significantly with particle size, with field and laboratory studies frequently demonstrating that organic carbon (OC) content increases with decreasing SSA particle size below 4 μm.^8,10,11^

Chemical aging can affect the composition of inorganic ions in SSA through processes such as chlorine depletion^12,13^ and formation of secondary inorganic ions like nitrate and sulfate.^13–16^ OC in SSA can be chemically modified after emission due to environmental processes such as oxidation and heterogeneous reactions.^8,17–19^ The oxidation of volatile organic compounds (VOC) by atmospheric oxidants, such as hydroxyl radicals, results in the formation of semi-volatile and low-volatile compounds that can partition to the particle phase.^8,18^ Gases such as dimethyl sulfide (DMS), isoprene, and a range of volatile inorganic and organic compounds are emitted directly from the ocean into the atmosphere, where they undergo oxidation and can contribute to the formation of secondary marine aerosols (SMA).^20–22^ Aerosol radiative properties, hygroscopicity, and cloud droplet nucleation are affected by the chemical composition of SSA and can evolve as aerosols undergo chemical transformations.^23,24^

Organosulfates (OS), containing a sulfate ester functional group, can arise from either primary or secondary sources in the marine environment. Primary emissions of OS include alkyl sulfates (i.e., dodecyl sulfate) used as anionic surfactants and detergents that occur in wastewater and coastal marine environments.^25–27^ These surface-active species can be enriched in the sea surface microlayer and transferred into the atmosphere by bubble bursting on the ocean surface.^2^ After emission, nascent SSA can undergo heterogeneous and/or photochemical reactions in the atmosphere to form additional OS.^28^ For example, long-chain unsaturated fatty acids produced through enzymatic degradation of algal biomass can be oxidized to form OS in the presence of acidic sulfate.^29^ The marine environment offers conditions that are conducive to the formation of OS, such as acidic seed particles,^30^ sulfate aerosols arising from the oxidation of DMS produced by marine life,^24,31^ and biogenic VOCs like isoprene and monoterpenes.^32,33^ For example, isoprene-derived OS can form via the reactive uptake of isoprene epoxydiols (IEPOX) on acidic sodium sulfate.^34^ 2-Methyltetrol sulfates can be further oxidized, converting primary alcohols into aldehydes and subsequently carboxylic acids that undergo ring-closing reactions.^35–37^

Fatty acids have been previously identified as a prevalent organic component in nascent SSA, with saturated and unsaturated fatty acids accounting for up to 20% of the OC in submicron particles.^8,38,39^ These compounds are naturally occurring surfactants that accumulate at the air–water interface and transfer into SSA, particularly fine SSA particles.^40–43^ In the atmosphere, fatty acids are subject to oxidation by atmospheric oxidants like ozone and hydroxyl radicals.^44–46^ As aerosol ages, the prevalence of shorter-chain fatty acids has been shown to increase due to the oxidation of longer-chain unsaturated fatty acids,^47–50^ which enhances the hygroscopicity of the aerosol and its ability to uptake water.^45,51^

Alkyl amines, released into the atmosphere through various natural processes such as emissions from phytoplankton and the decomposition of organic matter, make a substantial contribution to the pool of organic nitrogen.^52,53^ Their abundance in SSA is particularly highlighted in submicrometer particles, where alkyl amines can contribute up to 11% of the marine aerosol mass.^54,55^ The ability of alkyl amines to form ammonium salts with atmospheric acids plays a significant role in their atmospheric behavior, facilitating the formation of secondary aerosol mass.^52,56,57^ This process can be influenced by the chemical aging of SSA, which can increase particle acidity and favor the gas-to-particle partitioning of alkyl amines.^57^ Consequently, the presence of alkyl amines and their transformations in the atmosphere underscore their importance in atmospheric chemistry, especially in the context of acidity and particle-phase partitioning.

In this study, we examine bulk and molecular changes that nascent SSA undergoes in the presence of coemitted gas-phase species during simulated atmospheric aging (4–5 days). SSA was generated in the laboratory from coastal seawater that underwent a phytoplankton bloom. Chemical measurements included inorganic ions, OC, and organic species, including OS, fatty acids and their derivatives, and alkyl amines. These measurements reveal the molecular modifications to nascent SSA as it evolves upon aging and enhance our understanding of the chemical transformations of SSA in the atmosphere.

## 2 Experimental Method

### 2.1 Collection of SSA

Nascent SSA was generated from natural seawater via breaking waves generated in a 33 m glass wave channel at Scripps Institution of Oceanography, University of California, San Diego during the Sea Spray Chemistry and Particle Evolution (SeaSCAPE) study from June 29 to August 9, 2019. Details about the SeaSCAPE experiment, including comprehensive descriptions of the wave channel, seawater collection process, bloom initiation, SSA formation, and sampling channels, are described by Sauer et al.^16^ Briefly, seawater was collected from the Scripps Pier in La Jolla, California, filtered to remove debris and large particulates, and transported to the wave channel (11,800 L). Consistent with previous mesocosm studies, this experiment initiated a phytoplankton bloom by adding nutrients to the seawater, stimulating the microbial loop.^58,59^ Phytoplankton biomass was monitored by measuring the daily average chlorophyll-*a* concentrations throughout the experiment.^16^ SSA samples were collected before, during, and after the phytoplankton bloom, with samples combined to examine pre-bloom, peak-bloom, and post-bloom conditions.

SSA samples were collected from three channels (A, B, C). Channel A represented “nascent SSA” directly collected from the headspace of the wave flume. Channel B represented “aged SSA” formed by chemical aging of nascent SSA generated near wave breaking in the wave channel headspace. Aging was conducted with a potential aerosol mass-oxidative flow reactor (PAM-OFR; Aerodyne Inc.) that simulated atmospheric photochemical aging conditions equivalent to approximately 4–5 days.^16^ Channel C also represented “aged SSA” that was collected from the wave channel headspace away from wave breaking. Channel C utilized an isolated sampling vessel to isolate gases produced from seawater under clean conditions, minimizing contamination from ambient air and off-gassing from wave channel materials as described by Sauer et al.^16^ Gases and nascent SSA produced within the isolated vessel were then oxidized in another OFR.^16^ In comparison to channel B, channel C has a larger relative influence from SMA formation, as discussed in Section 3.3.

In channels A and B, nascent and aged SSA were collected over a period of approximately 48 h using a five-stage Sioutas Personal Cascade Impactor (PCIS, SKC model 225-370, 50% cutoff aerodynamic diameters: >2.5, 1.0, 0.5, 0.25, and <0.25 μm) at 9 L min^–1^ at relative humidities ranging from 74% to 96% (Figure S1). The first four stages of the Sioutas Impactor used 25 mm aluminum foil substrates, while a 37 mm quartz fiber filter (QFF, PALL Life Sciences) was used for the after filter. All substrates were prebaked at 550 °C for 18 h prior to sampling. Total suspended particles (TSP) in channel C were collected over a period of approximately 48 h using a single-stage holder (PALL Life Sciences model 1235) at 3 L min^–1^ on a 47 mm quartz fiber filter (QFF, PALL Life Sciences) (Figure S1). One field blank was collected for every five SSA samples. All collected samples were stored in aluminum foil (prebaked at 550 °C for 5.5 h) lined Petri dishes at −20 °C under dark conditions until extracted.

### 2.2 Analysis of Inorganic Ions

The concentrations of cations and anions in nascent (channel A) and aged (channel B) SSA were quantified from the ultrapure water extract using high-performance ion-exchange chromatography with conductivity detection, as previously described in Jayarathne et al.^60^ Samples from channel C were not analyzed for ions due to the allocation of filter samples for organic speciation. The analytical uncertainty in inorganic ion concentrations was propagated by accounting for the relative errors in extraction efficiency and the relative error in field blanks. The relative error in extraction efficiency was calculated as the measurement multiplied by the difference between the observed and expected concentrations of the quality control samples. For cations, this difference ranged from 5% to 10%, and for anions, it ranged from 8% to 17%. The relative error in field blanks was determined by using the standard deviation of field blanks.

### 2.3 Analysis of Organic Carbon

Total organic carbon (OC) measurements were conducted for channels A, B, and C over three sampling periods (July 26–27, August 1–2, and August 9–10 in 2019), representing different biological activities in seawater. OC in both QFF and Al substrates was measured using a thermal-optical analyzer via NIOSH method 5040, with the maximum temperature of 870 °C in the helium stage (Sunset Laboratories).^61^ For the QFF substrates, a thermal-optical transmittance method was used for the ECOC split; EC was not detected. For Al substrates, an ECOC split was not assigned due to the inability to perform optical correction, and based on the QFF analysis, only OC was assumed to be present. For stages A through D, a subsample comprising one-eighth (1/8) of the filter area was used, while for stage E, a 1.00 cm^2^ subsample was employed for OC analysis. All measurements were field blank subtracted. The analytical uncertainty of OC concentration was propagated by accounting for the relative uncertainty in instrumental analysis (calculated from the summation of 5% of the measurement and instrumental limit of detection) and the relative uncertainty in field blanks, calculated from the standard deviation of the field blanks.

### 2.4 Extraction of Organic Species, Separation, Quantitative and Qualitative Analysis

Extraction of organic species followed Hettiyadura et al.,^37^ in which substrate-deposited SSA samples, collected from channels A, B, and C, and field blanks were submerged in 10.0 mL of acetonitrile and ultrapure water (95:5, by volume) and extracted by ultrasonication (60 sonics min^–1^ for 20 min.^37^ Ultrapure water was prepared on-site (Thermo, Barnstead EasyPure-II; 18.2 MΩ·cm resistivity, with total OC <40 μg L^–1^). Acetonitrile (Optima-LC/MS Grade, Fisher Scientific) was used without further purification. Extracts were then filtered through polypropylene membrane syringe filters (0.45 μm pore size, Puradisc 25PP, Whatman). The volume of the filtrate was reduced up to 500 μL under an ultrahigh purity nitrogen gas stream (≤5 psi) at 50 °C using an evaporating system (Turbovap LV, Caliper Life Sciences). The obtained extracts were blown to dryness at 50 °C using a microscale nitrogen evaporating system (Reacti-Therm III TS 18824 and Reacti-Vap I 18825, Thermo Scientific) after transferring them into 1.5 mL LC vials. Then the extracts were reconstituted with acetonitrile and ultrapure water (95:5, by volume) to a final volume of 70 μL.^37^

Seven standards were used for the quantification and semi-quantification of OS present in SSA. Methyl sulfate, ethyl sulfate, and dodecyl sulfate standards were purchased from Sigma-Aldrich. Glycolic acid sulfate, hydroxyacetone sulfate, acetoin sulfate, and benzyl sulfate were synthesized as potassium salts (<95% purity) following the procedure described in Hettiyadura et al.^37^ An ultraperformance liquid chromatography (UPLC) interfaced to a triple quadrupole mass spectrometer (Xevo TQ-S cronos, Waters) with negative electrospray ionization ((−) ESI), operating in multiple reaction monitoring (MRM) mode, was used for the quantification of OS by following the optimized conditions explained in Hettiyadura et al.^37^ An ethylene-bridged hybrid (BEH-amide) column (2.1 × 100 mm, 1.7 μm particle size; ACQUITY UPLC Waters) was used for the separation. Elution of the analytes was achieved using a mobile phase gradient, which consists of an organic eluent of ammonium acetate buffer (10 mM, pH 9) in acetonitrile and ultrapure water (95:5, by volume) and an aqueous eluent of ammonium acetate buffer (10 mM, pH 9) in ultrapure water. Methyl sulfate, ethyl sulfate, dodecyl sulfate, acetoin sulfate, benzyl sulfate, hydroxyacetone sulfate, and glycolic acid sulfate were quantified using authentic standards. For semi-quantitation of other OS that fragmented to the bisulfate anion (*m*/*z* 97) and eluting prior to 4 min hydroxyacetone sulfate was used as a surrogate standard. For those retaining more than 4 min, glycolic acid sulfate was used. For the semi-quantitation of OS that fragmented only to the sulfate radical anion (*m*/*z* 96), methyl sulfate was used as a surrogate quantification standard. Data were acquired and analyzed using MassLynx (Waters Inc.; version 4.1).

OS included compounds that fragmented to the bisulfate anion (*m*/*z* 97) and/or a sulfate radical anion (*m*/*z* 96) using HILIC–TQ-S Cronos in the precursor ion mode scanning masses within the range of 100–400 Da. A cone voltage of 28 V and a collision energy of 16 eV were used for precursors of *m*/*z* 97. A cone voltage of 42 V and a collision energy of 20 eV were used for precursors of *m*/*z* 96. The formulas of target compounds were identified via ultraperformance liquid chromatography coupled to a quadrupole Exactive Orbitrap mass spectrometer (UPLC-Q-Exactive-Orbitrap-MS) with a heated electrospray ionization source (HESI). The following optimized ionization conditions were used in the negative mode: spray voltage of 3.5 kV, capillary temperature of 270 °C, vaporizer temperature of 438 °C, S-lens frequency of 70 Hz, sheath gas flow rate of 54 arbitrary units (au), auxiliary gas flow rate of 14 au, and sweep gas flow rate of 0. For the analysis, two acquisition modes, full scan (FS) and data-dependent MS2 (dd-MS2), were used. Resolving power of 70 000, automatic gain control (AGC) of 1 × 10^6^, and maximum injection time of 200 ms were the full scan optimized settings. The mass range was 50–400 Da. Resolving power of 17 500, AGC of 1 × 10^5^, maximum injection time of 50 ms, and normalized collision energy (NCE) of 30 eV were the optimized dd-MS2 settings. Data were acquired and analyzed using MassLynx (Waters Inc.; version 4.1) and Xcalibur (Thermo; version 4.2) software. Molecular formulas were assigned considering C_0–30_, H_0–60_, N_0–10_, O_0–10_, S_0–6_, odd and even electron states, and a maximum error of 1 mDa.

### 2.5 Qualitative Analysis of Fatty Acids and Alkyl Amines

The targeted analysis of individual molecules was performed in both negative and positive modes using Q-Exactive-Orbitrap-MS to identify fatty acids and alkyl amines, respectively. The targeted analysis was based on an inclusion list, which was compiled from previous literature. This analysis utilized the same optimized conditions described above. Data were acquired using Xcalibur (Thermo; version 4.2) and analyzed using Compound Discoverer 3.3 (Thermo Scientific).

## 3 Results and Discussion

### 3.1 Inorganic Ions in Nascent and Aged SSA

The inorganic ion composition of nascent SSA primarily consists of ions, such as Na^+^, Cl^–^, SO_4_^2–^, Ca^2+^, and Mg^2+^ (Figure S2). Among these, Na^+^ and Cl^–^ are the most significant contributors to particle mass >0.25 μm, accounting for approximately 31% and 55% of the measured PM mass, calculated as the sum of measured cations, anions, and OC. Mg^2+^ and SO_4_^2–^ contributed 4% and 7% of the PM mass, respectively, while PO_4_^3–^, NO_3_^–^, and NH_4_^+^ contributed <1%. The mass concentration of salt and its contribution to particle mass both decreased as particle size decreased from 2.5 to <0.25 μm. Because absolute concentrations of inorganic ions indicated aerosol mass losses in channel B compared to channel A (Figure S2) particularly for supermicrometer particles, ion ratios were used to examine relative changes in SSA composition.

To investigate the impact of chemical aging, the ratios of inorganic ions to Na^+^ were determined for both nascent and aged SSA (in channels A and B, respectively). Na^+^ was used as a reference because of its abundance in SSA and the expectation that its concentration will not be altered by chemical aging. Oxidization generally increased secondary (non salt) inorganic ions relative to nascent SSA, as demonstrated by sulfate (SO_4_^2–^), phosphate (PO_4_^3–^), nitrate (NO_3_^–^), and ammonium (NH_4_^+^) (Figure 1a−d). These enhancements are expected to result from the oxidation of sulfur, phosphorus, and nitrogen-containing gaseous precursors that are produced through the life cycles of marine microbes.^21,22^ Sulfate, for instance, can be produced from the oxidation of sulfur dioxide, dimethyl sulfide, and other gas-phase sulfur species like hydrogen sulfide and dimethyl disulfide.^21,62–64^ The SO_4_^2–^ to Na^+^ ratio in aged SSA increased across all particle sizes compared to nascent SSA. This increase was on the order of 20–30% in particles >0.25 μm, but reached a factor of 7 in particles <0.25 μm. The enhancement of SO_4_^2–^ to Na^+^ across the SSA sizes studied suggests secondary SO_4_^2–^ formation across the range of particles studied.^62^ Other inorganic ions increased consistently in aged SSA compared to nascent SSA across all measured particle sizes, with PO_4_^3–^ enhanced by factors of 7–31, NO_3_^–^ by 4–77, and NH_4_^+^ by 13–24. The increase in NH_4_^+^ to Na^+^ in aged SSA is indicative of an increase in the SSA acidity level due to chemical aging. The molar ratios of NH_4_^+^ to anions from non sea salt SO_4_^2–^, and NO_3_^–^ also indicated that aged SSA was more acidic relative to nascent SSA. Meanwhile, the chloride (Cl^–^) to Na^+^ ratio showed no significant difference between nascent and aged SSA across the five size fractions examined (Figure 1e) indicating that there was no appreciable chloride depletion, which was observed previously in the aging of SSA.^12^

**Figure 1:**
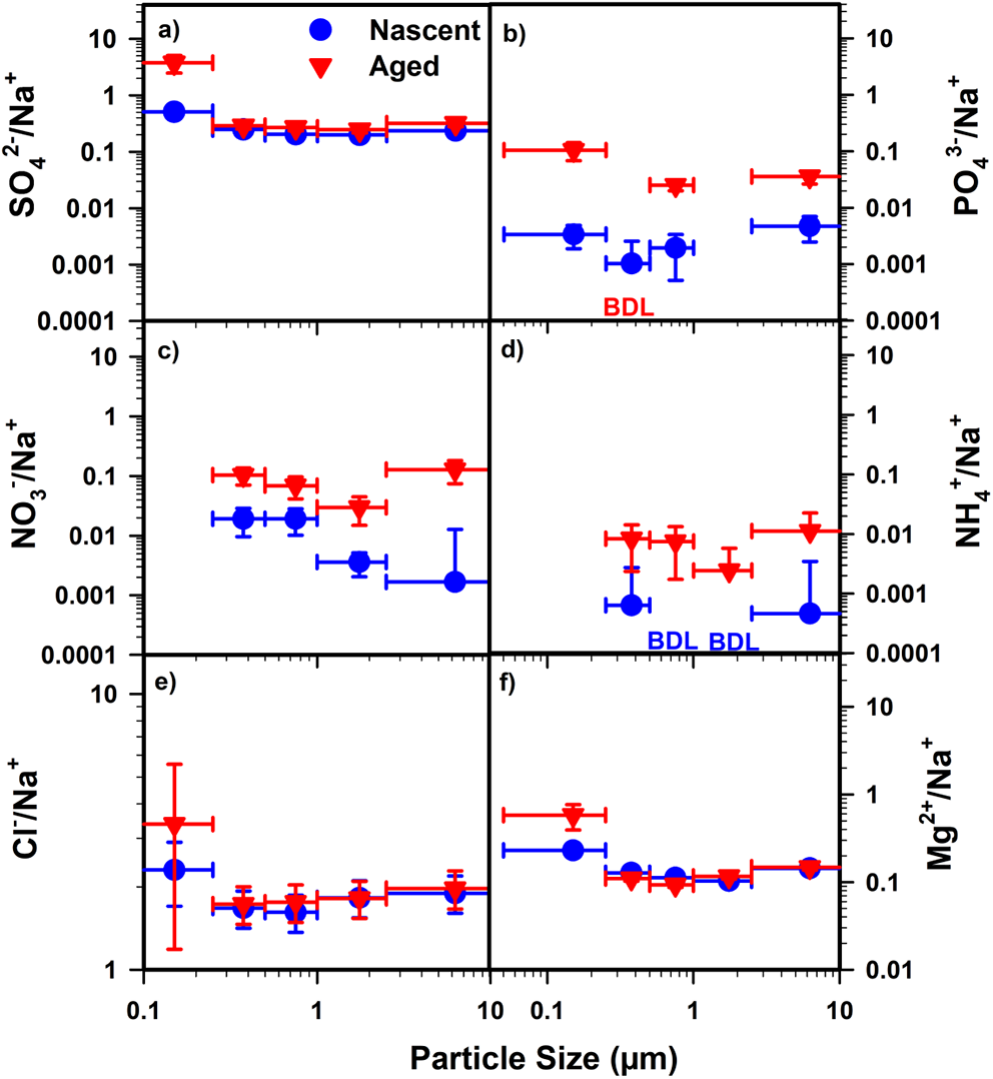
(a) SO_4_^2–^/Na^+^, (b) PO_4_^3–^/Na^+^, (c) NO_3_^–^/Na^+^, (d) NH_4_^+^/Na^+^, (e) Cl^–^/Na^+^ (f) Mg^2+^/Na^+^ mass ratios for nascent and aged SSA on August 1–2, 2019 (peak of the bloom). “BDL” denotes measurements below the detection limit.

### 3.2 Organic Carbon in Nascent and Aged SSA

OC in nascent and aged SSA was measured at different stages of biological activity in seawater: pre-bloom (July 26–27), peak of the bloom (August 1–2), and post-bloom (August 9–10), respectively. The OC to Na^+^ ratio in samples of nascent SSA increased with decreasing particle size, as also demonstrated by previous studies.^8,10,11^ During the bloom’s peak on August 1–2, the OC to Na^+^ ratio in nascent SSA for particles <0.25 μm increased by up to a factor of 600 compared to particle sizes >0.25 μm (Figure 2). The size-dependent trend, in which the OC to Na^+^ ratio increases as particle size decreases, along with a significant enhancement of the OC to Na^+^ ratio in particle size <0.25 μm, was consistent across other nascent SSA samples collected during the pre-bloom and post-bloom periods (Figure S3). In the pre-bloom phase, the enhancement of OC to Na^+^ in particle size <0.25 μm compared to particle sizes >0.25 μm was up to a factor of 1 × 10^2^, while in the post-bloom phase it reached 7 × 10^3^. These observations indicate that smaller particles in nascent SSA consistently have a higher OC to Na^+^ ratio compared to larger particle sizes and that the extent of this OC enrichment varies across different levels of biological activity in the seawater. Previous studies have demonstrated that OC levels in SSA increase with increased biological activity in seawater.^5,11,58^ The greater enhancement of the OC to Na^+^ ratio in particles <0.25 μm during the post-bloom phase, compared to both pre-bloom and peak bloom periods, suggests that biological processes, such as the release of organic matter from phytoplankton, bacterial degradation of cellular material, and the secretion of extracellular compounds by bacteria intensify OC transfer to nascent SSA in the post-bloom phase.^58–66^

**Figure 2:**
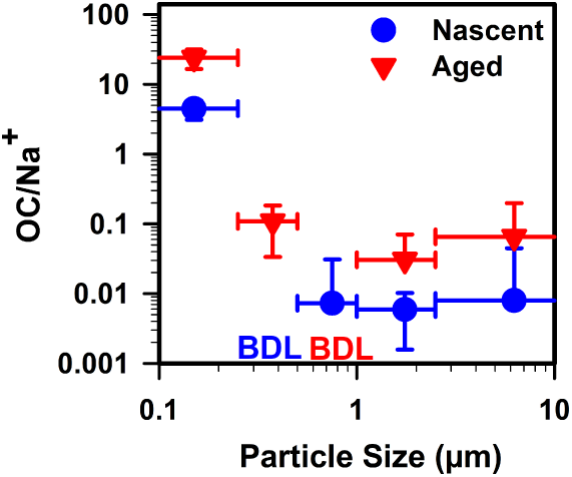
Organic carbon (OC)/sodium (Na^+^) ratios for nascent and aged SSA on 1–2 August 2019 (peak of the bloom). “BDL” denotes measurements below the detection limit.

Chemical aging further increased the OC to Na^+^ ratio in aged SSA relative to nascent SSA in all size ranges, with particles <0.25 μm showing an increase by up to a factor of 5 (Figure 2). Collocated measurements revealed the appearance of a nucleation mode in the aerosol distribution of aged SSA, indicating a new particle formation.^16^ However, our analysis could not definitively discern between new particle formation and particle growth, given that the smallest measured particle size was OC <0.25 μm, encompassing particles from both processes. For particles >0.25 μm we anticipate that the predominant increase in OC results from particle growth driven by the oxidation of VOC, reducing their volatility and causing them to condense onto existing particles. The bulk composition of nascent and aged SSA supports the hypothesis that chemical reactions of SSA modify its composition and properties in size-dependent manners.

The increase in OC in the particle size <0.25 μm co-occurs with the enrichment of Mg^2+^ in nascent and aged SSA particles (Figure 1f). The Mg^2+^ to Na^+^ ratio increased by up to a factor of 2 in particle size <0.25 μm compared to particles >0.25 μm in nascent SSA. The observed enrichment of Mg^2+^ in this particle size range may result from the complexation of Mg^2+^ by organic ligands that enhances its transfer into nascent SSA.^8,9,11^

### 3.3 Quantification and Identification of OS in SSA

Next, we test the hypothesis that OS species contribute to the OC formed in SSA upon the aging of the gases and SSA particles emitted from the wave channel. The conducive environment for OS formation in nascent SSA is primarily facilitated by the abundant presence of inorganic sulfate and the acidic nature of nascent SSA, which quickly reaches a pH of around 2 for submicron particles after being ejected from the ocean.^30^ The OS concentration in SSA was evaluated using 23 compounds, of which 7 were quantified using authentic standards and 16 were semi-quantified (Table S1). The average OS mass concentrations and mass fractions in PM_1.0_ nascent, aged SSA (in channels A and B), and in channel C were assessed during the phytoplankton bloom (August 3–4) and post-bloom (August 7–8). The average OS mass concentrations in PM_1.0_ nascent (channel A), PM_1.0_ aged SSA (channel B), and TSP in channel C were assessed during the phytoplankton bloom (August 3–4) and post-bloom (August 7–8) period as the average of consecutive days. Mass fractions of OS in OC were calculated as the sum of the individual OS concentrations relative to OC. Nascent SSA was observed to have an average OS concentration of 1.8 ng m^–3^, contributing 0.18% to OC. Dodecyl sulfate, an anthropogenic surfactant that is expected to enter nascent SSA from runoff in coastal seawater, had the strongest OS signal in nascent SSA and was largely responsible for higher OS concentrations in nascent SSA (Table S1). Aged SSA in channel B had lower OS concentrations at 0.3 ng m^–3^, contributing only 0.01% to OC, suggesting that aging either degraded primary OS and/or that OC formed by aging had low OS fractions. Channel C, containing aged TSP and SMA, had the highest OS concentration of 3.7 ng m^–3^ with a 0.95% contribution to OC. In particular, the contribution of isoprene-derived OS to OC increased from 0.01% in nascent SSA to 0.65% in channel C, while monoterpene-derived OS increased from 0.003% in channel A to 0.08% in channel C, indicating the formation of isoprene- and monoterpene-derived OS to OC in channel C. In all three channels, OS made minor contributions to SSA mass, and the relative concentrations spanned nearly 3 orders of magnitude.

Because the 23 quantified OS species accounted for only a small fraction of the OC, we employed precursor ion scans to obtain a broader view of the OS present in PM_1.0_ SSA. Characteristic product ions of OS, including bisulfate anions (HSO_4_^–^ at *m*/*z* 97) and sulfate radical anions (SO_4_^–·^ at *m*/*z* 96) formed under negative mode electrospray ionization conditions, were used for the identification of OS in nascent and aged SSA.^37,67–69^ Figure S4 illustrates the MS signals corresponding to the major OS observed in nascent, aged SSA (channels A and B), and in channel C. Because bisulfate is an even-electron species and is more stable than the odd-electron sulfate radical, it had a much stronger MS/MS signal (by approximately 20×). Tentative structures, VOC precursors, monoisotopic masses of observed OS in aged SSA, and their relative contributions to the total HSO_4_^–^, *m*/*z* 97 precursor ion signal were recorded in Table 1.

**Table 1:**
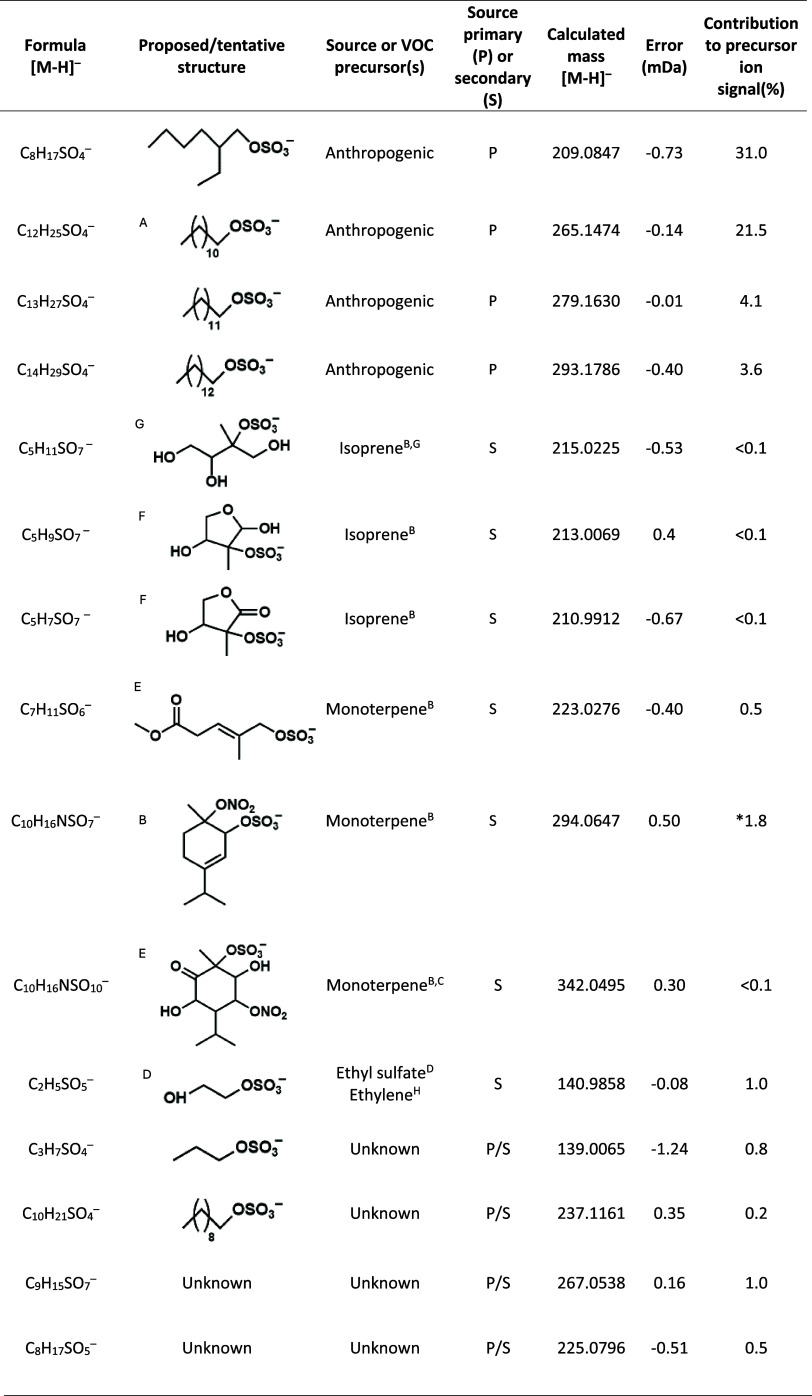
OS Was Identified in PM_1.0_ Aged SSA with the Strongest Contributions to the Bisulfate/Sulfate Anion Radical Precursor Ion Signal[Table-fn tbl1fn1]

A Hettiyadura et al.,^67^;

* Contribution to *m/z* 96 precursor ion signal (%).

B Surratt et al.,^70^;

C Iinuma et al.,^71^;

D Kwong et al.,^72^;

E Yassine et al.,^73^;

F Hettiyadura et al.,^37^;

G Surratt et al.,^74^;

H Hughes et al.,^69^

Figure 3 illustrates concentrations of OS in PM_1.0_ nascent (channel A), PM_1.0_ aged SSA (channel B), and TSP in channel C, which contains SMA from oxidized VOCs and aged SSA. OS were classified into three categories in Figure 3: isoprene-derived OS (green bars), monoterpene-derived OS (red bars), and low molecular weight OS whose structures are relatively well characterized (purple bars). Comparative analysis of OS concentrations in nascent and aged SSA (in channels A and B, respectively) revealed that some OS have increased concentrations, while others have decreased concentrations in aged SSA relative to nascent SSA across all three categories. The increased OS concentration indicates the formation of these OS within the OFR as a result of chemical aging. In contrast, the decreased concentration suggests degradation or further oxidation of OS through heterogeneous reactions inside the OFR. These findings highlight that chemical aging has the capability to form OS as well as to further oxidize them.

**Figure 3:**
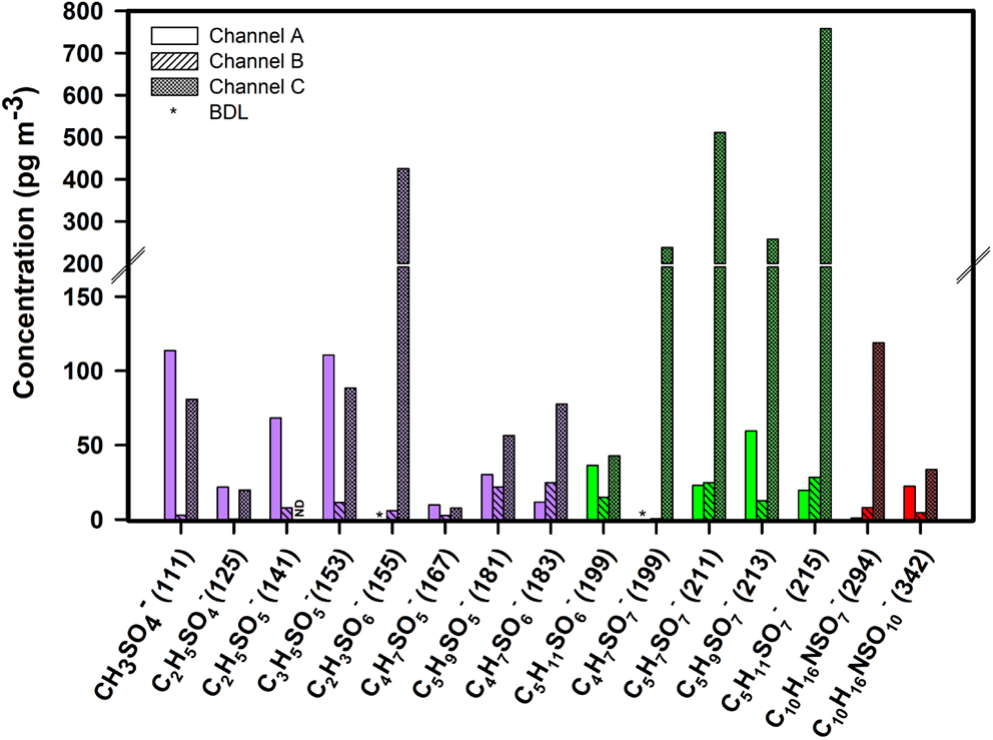
Concentrations of quantified organosulfates in PM_1.0_ nascent SSA (channel A), PM_1.0_ aged SSA (channel B), and in TSP containing SMA and aged SSA (channel C) collected at Scripps Institution of Oceanography, University of California San Diego on August 3–4, 2019 (peak of the bloom). Organosulfates are color-coded as purple for low molecular weight species, green for isoprene-derived, and red for monoterpene-derived. Asterisks indicate values below the detection limit (BDL); ND signifies not detected.

#### 3.3.1 Alkyl Sulfates

The four strongest signals correspond to alkyl OS that were identified as ethylhexyl sulfate (C_8_H_17_SO_4_^–^, *m*/*z* 209.0847), dodecyl sulfate (C_12_H_25_SO_4_^–^, *m*/*z* 265.1474), tridecyl sulfate (C_13_H_27_SO_4_^–^, *m*/*z* 279.1630), and myristyl sulfate (C_14_H_29_SO_4_^–^, *m*/*z* 293.1786). Among these alkyl sulfates, only dodecyl sulfate was quantified, and its concentrations in each channel are reported in Table S1. The presence of these alkyl OS in SSA indicates anthropogenic influences on the coastal environment because these compounds are common surfactants in hygiene and cleaning products and are likely to come from primary sources such as surface and wastewater runoff into the ocean.^25–27^ C_3_H_7_SO_4_^–^ (*m*/*z* 139.0065) and C_10_H_21_SO_4_^–^ (*m*/*z* 237.1161) were also identified as alkyl sulfates with relatively low contributions to the bisulfate precursor ion scan (0.8% and 0.2%, respectively).

Evidence of chemically aged alkyl sulfates was observed via the detection of C_8_H_17_SO_5_^–^ (*m*/*z* 225.0796) with a 0.5% contribution to the precursor ion scan. The literature reports on oxidation mechanisms of alkyl sulfates indicate that they undergo functionalization via hydrogen abstraction followed by hydroxyl addition.^75^ Given these pathways, C_3_H_7_SO_5_^–^ is likely formed from the heterogeneous OH oxidation of ethylhexyl sulfate, which contributed 31% to the *m*/*z* 97 precursor ion signal. Additionally, the first-generation oxidation products of dodecyl sulfate, tridecyl sulfate, and myristyl sulfate with formulas C_12_H_25_SO_5_^–^ (*m*/*z* 281.1423), C_13_H_27_SO_5_^–^ (*m*/*z* 295.1579), and C_14_H_29_SO_5_^–^ (*m*/*z* 309.1736) were detected with contributions to the *m*/*z* 97 precursor ion signal <0.1%.

#### 3.3.2 Isoprene-Derived Organosulfates

The concentrations of 2-methyltetrol sulfate (C_5_H_11_SO_7_^–^, *m*/*z* 215.0225), sulfate esters of methyldihydroxylactone (C_5_H_7_SO_7_^–^, *m*/*z* 210.9912), and 2-methylglyceric acid sulfate (C_4_H_7_SO_7_^–^, *m*/*z* 198.9912) increased upon aging in channel B, which could be due to the formation of these compounds by oxidizing isoprene in the presence of acidic sulfate. For example, 2-methyltetrol sulfate (C_5_H_11_SO_7_^–^, *m*/*z* 215.0225), an OS formed via acid-catalyzed nucleophilic addition of sulfate to isoprene epoxides (IEPOX)^74^ had concentrations of 19.6 and 28.5 pg m^–3^ in nascent and aged SSA, respectively, suggesting that it formed in the OFR as a result of chemical aging. In contrast, sulfate esters of cyclic methyltrihydroxyaldehyde hemiacetal (C_5_H_9_SO_7_^–^, *m*/*z* 213.0069) and (C_5_H_11_SO_6_^–^, *m*/*z* 199.0276) showed lower concentrations in aged SSA compared to nascent SSA, likely due to the degradation of those compounds in the OFR through further oxidation. It has been shown in previous studies that OS can be further oxidized and fragmented in the atmosphere.^35–37^ For example, 2-methyltetrol sulfate (C_5_H_11_SO_7_^–^, *m*/*z* 215.0225) can undergo heterogeneous oxidation in the atmosphere, leading to the formation of sulfate esters of cyclic methyltrihydroxyaldehyde hemiacetal (C_5_H_9_SO_7_^–^, *m*/*z* 213.0069) and sulfate esters of methyldihydroxylactone (C_5_H_7_SO_7_^–^, *m*/*z* 210.9912) through oxidation to an aldehyde and carboxylic acid, respectively, followed by ring closure.^37^ Additionally, 2-methylglyceric acid sulfate (C_4_H_7_SO_7_^–^, *m*/*z* 198.9912) is identified as an OS formed by oxidizing 2-methyltetrol sulfate (C_5_H_11_SO_7_^–^, *m*/*z* 215.0225) and subsequent fragmentation by the further atmospheric oxidation.^35,75^ The oxidation of OS in the OFR is demonstrated by decreased concentrations of 2-methyltetrol sulfate (C_5_H_11_SO_7_^–^, *m*/*z* 215.0225) and increased concentrations of its oxidation products (*m*/*z* 210.9912, 213.0069, and 198.9912) in aged SSA. This effect was particularly strong in aged PM_2.5_ SSA particles (Figure S5), but less pronounced in aged PM_1.0_ SSA particles (Figure 3). Although there was not a decrease in 2-methyltetrol sulfate (C_5_H_11_SO_7_^–^, *m*/*z* 215.0225) concentration, the observed increase in sulfate esters of methyldihydroxylactone (C_5_H_7_SO_7_^–^, *m*/*z* 210.9912) and 2-methylglyceric acid sulfate (C_4_H_7_SO_7_^–^, *m*/*z* 198.9912) OS concentrations in PM_1.0_ aged SSA may have resulted from the further oxidation of 2-methyltetrol sulfate (C_5_H_11_SO_7_^–^, *m*/*z* 215.0225). Compared to other OS, isoprene-derived OS were greatly enhanced in channel C. The concentration of 2-methyltetrol sulfate (C_5_H_11_SO_7_^–^, *m*/*z* 215.0225) in channel C was nearly 40 times higher than its concentration in nascent SSA. This significant increase suggests effective gas-to-particle conversion of isoprene in the marine environment.

#### 3.3.3 Monoterpene-Derived Organosulfates

The nitroxy-OS, C_10_H_16_NSO_7_^–^ (*m*/*z* 294.0647) and C_10_H_16_NSO_10_^–^ (*m*/*z* 342.0495), are suggested to form from monoterpenes by photooxidation in the presence of NO_*x*_.^70–72^ C_10_H_16_NSO_7_^–^ (*m*/*z* 294.0647) exhibited higher concentrations in aged SSA compared to nascent SSA, indicating its formation in channel B through chemical aging. Moreover, C_10_H_16_NSO_7_^–^ (*m*/*z* 294.0647) exhibited enhanced concentration in channel C, with a concentration of 119 pg m^–3^, representing a 120-fold increase over its concentration in nascent SSA. Like isoprene-derived OS, monoterpene-derived OS can also undergo further oxidation in the atmosphere, leading to functionalized OS.^75^ The decreased concentration of C_10_H_16_NSO_10_^–^ (*m*/*z* 342.0495) in aged SSA may have resulted from this additional oxidation. Additionally, in the bisulfate precursor ion scan, an OS with the formula C_7_H_11_SO_6_^–^ (*m*/*z* 223.0271) was identified as a monoterpene oxidation product, formed in the presence of sulfate and NO_*x*_,^70^ contributing of 0.5% to the bisulfate ion signal.

#### 3.3.4 Fatty Acid-Derived Organosulfates

Fatty acid-derived OS (C_*n*_H_2*n*–1_SO_6_®: *m*/*z* 253.0746, 267.0902, 295.1215) were identified in the bisulfate precursor ion scan in aged SSA. These compounds are likely formed through the oxidation of hydroxylated and unsaturated fatty acids and subsequent reaction with sulfuric acid.^29^ A homologous series of C_6_–C_10_, C_12_–C_16_, and C_18_–C_19_ fatty acid-derived OS was identified in aged SSA, although their contribution to the total precursor ion signal is <0.1% (Table S2).

#### 3.3.5 Organosulfates from Unidentified Precursors

C_2_H_5_SO_5_^–^, *m*/*z* 140.9858 was tentatively identified as 2-hydroxyethyl sulfate. While this compound is known to form through the oxidation of ethyl sulfate,^72^ it has also been suggested that it could originate from the oxidation of ethylene (Table 1).^69^ Ethylene can be emitted from marine waters^76^ and undergoes reactive uptake, where ethylene oxide reacts via acid-catalyzed ring opening followed by the nucleophilic addition of sulfate.^69^

A previously unreported OS with elemental composition C_9_H_15_SO_7_^–^, *m*/*z* 267.0538 was qualitatively identified in the bisulfate precursor ion scan, contributing 1.0% of the signal (Table 1). The detection of this species in the marine environment suggests the existence of additional, yet unidentified, pathways or precursor compounds contributing to OS formation in the marine atmosphere.

### 3.4 Fatty Acids in Nascent and Aged SSA

Homologous series of saturated fatty acids (FA_sat_), unsaturated fatty acids (FA_unsat_), dicarboxylic acids, hydroxyl fatty acids, and oxo-fatty acids were identified in PM_2.5_ nascent, aged SSA (channel A and B), and in channel C (Table 2). Of the detected homologous series, FA_sat_ with C_8_–C_24_, exhibited the greatest MS signal in each channel, with C_18_ (stearic acid) contributing the largest signal. Mono-FA_unsat_ exhibited the next highest signal in nascent and aged SSA, with C_18:1_ (vaccenic acid) and C_16:1_ (palmitoleic acid) having the largest signals. The species that contributed to each fatty acid class are shown in Table 2. Carbon preference index (CPI) values for FA_sat_ in nascent, aged SSA, and channel C were all greater than 1, indicating the influence of biological activity within the wave flume system on FA_sat_ distributions. Fatty acids are generally susceptible to oxidation due to chemical aging. For example, as aerosol ages, long chain unsaturated fatty acids can break down into smaller chain fatty acids, increasing the prevalence of shorter-chain fatty acids, which could decrease the average molecular weight (AMW) values of unsaturated fatty acids.^45,50^ Although the literature provides evidence of the oxidation of long-chain unsaturated fatty acids and the formation of smaller-chain fatty acids,^47–50^ in our study there was neither a significant decrease in AMW nor CPI.

**Table 2:**
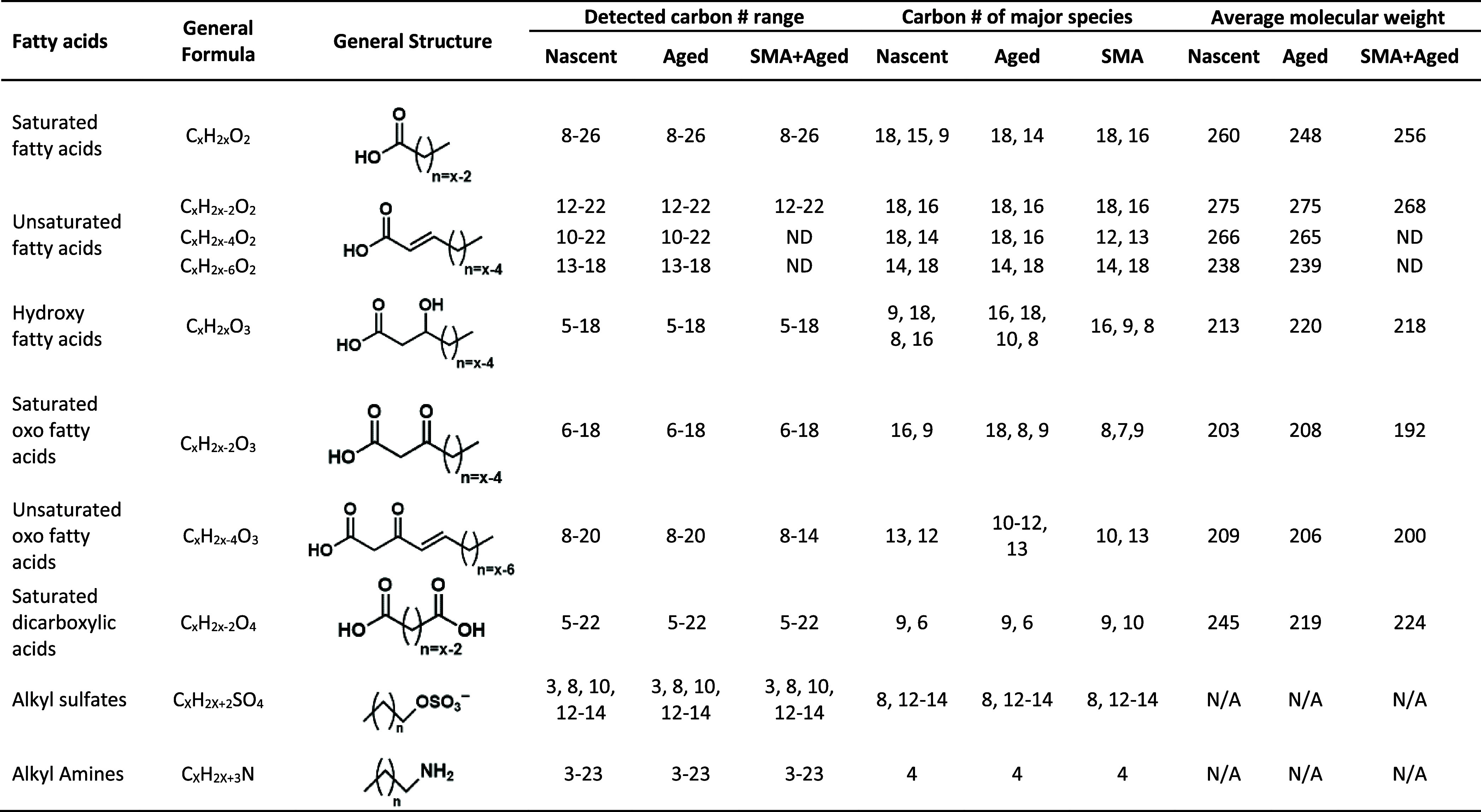
Observations of Fatty Acids, Alkyl Sulfates, and Alkyl Amines Identified in PM_2.5_ Nascent (Channel A) and Aged SSA (Channel B), and in SMA with Aged SSA (Channel C)

ND = not detected, AMW = Average molecular weight. The analyzed carbon # range for each compound class is as follows: saturated fatty acids (C_8_–C_26_), unsaturated fatty acids (C_10_–C_22_), hydroxy fatty acids (C_5_–C_21_), saturated oxo fatty acids (C_5_–C_18_), unsaturated oxo fatty acids (C_8_–C_22_), saturated dicarboxylic acids (C_3_–C_22_), alkyl amines (C_3_–C_25_).

### 3.5 Alkyl Amines in Nascent and Aged SSA

A homologous series of alkyl amines, expected to be present in SSA as ammonium salts, ranging from C_3_–C_23_, was detected in PM_1.0_ nascent, aged SSA (channel A and B), and in channel C (Table 2 and Figure S7). In addition to this homologous series, monomethylamine (MMA) and dimethylamine (the C_1_ and C_2_ homologues) are typically expected in SSA,^53^ but were below the selected MS mass range. Among the detected alkyl amines, diethylamine (DEA) exhibited the highest MS signal in nascent, aged SSA, and channel C. The presence of DEA in nascent SSA indicates its direct transfer from seawater to SSA. In aged SSA, the MS signal of DEA increased relative to that of nascent SSA. DEA’s transfer to the particulate phase is enhanced by acid–base reactions that form secondary ammonium ions from amines.^77,78^ Previous laboratory studies have shown that gaseous amines, including DEA, are irreversibly taken up into a sulfuric acid solution, mimicking acidic aerosol particles, with DEA having the highest uptake coefficient.^78^ The higher acidity in aged SSA is supported by its higher SO_4_^2–^ to Na^+^ and NO_3_^–^ to Na^+^ ratios compared to nascent SSA, which would facilitate the uptake of DEA. Additionally, alkyl amines can originate from chemical transformations induced by photooxidation and ozonolysis during the chemical aging process.^52,77,79^ These findings highlight that multiphase reactions contributing to chemical aging alter the molecular composition of SSA.

### 3.6 Chemical Changes to SSA upon Aging and Related Aerosol Properties

Upon aging for 4–5 days of simulated atmospheric aging, the composition of nascent SSA increased in the relative amount of OC and secondary inorganic ions, particularly SO_4_^2–^, NO_3_^–^, and NH_4_^+^ (Figure S2). Among these, OC and SO_4_^2–^ exhibited the greatest increases in particles <0.25 μm. Organic speciation of nascent and aged SSA revealed the uptake of semi-volatile alkyl amines and the formation of some OS upon aging. On a molar scale, the secondary sulfate formed in the OFR exceeded the concentration of OS by a factor of approximately 40 in PM_1.0_, indicating the predominance of chemical transformations of inorganic salts over OS. These results pertain to SSA generated from coastal, biologically active seawater. In different oceanic environments, varying levels of dissolved and particulate OC in seawater are expected to yield varying compositions of nascent and aged SSA OC.

Collocated measurements conducted during the SeaSCAPE campaign utilizing atomic force microscopy on single particles indicated that aging in channel B increased water uptake and hygroscopicity in particles with core–shell morphology relative to nascent SSA.^23^ In particular, aged SSA core–shell particles (particle size 0.32–0.60 μm) showed higher volume-equivalent growth factors (GF, 1.2–1.7) and hygroscopicity parameters (*κ*_Mix_, 0.5 ± 0.4) compared to nascent SSA (1.2–1.4, 0.3 ± 0.2, respectively).^23,80^ While changes to hygroscopicity are expected to be driven by inorganic salts formed upon aging, modifications to OC co-occur and can introduce more oxygenated functional groups with greater hygroscopicity.^20,81^ While OS concentrations are low, the surfactant properties of alkyl sulfates cause them to reside at particle surfaces, where they can decrease surface tension and facilitate water uptake at lower relative humidities.^82–84^ In the OFR aging experiment, the increases in sulfate and OC in aged SSA were greatest in particles <0.25 μm, so this size range is expected to experience the largest increase in water uptake. Collocated measurements in SeaSCAPE also indicated that the increased hygroscopicity of aged SSA can alter its phase, with core–shell particles in nascent SSA shifting from solid/semi-solid phase states to semi-solid/liquid particles upon aging.^23,85^ Phase state changes also impact aerosol light scattering and CCN activity, with aged liquid-phase SSA showing greater CCN activity than solid-phase nascent SSA.^86,87^ Enhanced CCN activity can extend cloud lifetime, increase cloud albedo, and reduce precipitation.^88–90^

## Supplementary Material



## Data Availability

The data set supporting this manuscript is hosted by the University of California, San Diego (UCSD) Library Digital Collections (10.6075/J0VM4CMJ).

## Notes

Schematic diagram of sampling nascent SSA (channel A), aged SSA (channel B), and SMA with aged SSA (channel C) (Figure S1), organic carbon (OC) and inorganic ion composition for (a) nascent and (b) aged SSA on 1–2 August 2019 (peak of the bloom) (Figure S2), organic carbon (OC)/sodium (Na^+^) ratios for nascent and aged SSA on (a) 26–27 August (b) 9–10 August 2019 (pre bloom and post bloom) (Figure S3), mass spectra corresponding to precursor ion scans of (A) bisulfate anion (*m*/*z* 97), and (B) sulfate ion radical (*m*/*z* 96) in PM_1.0_ nascent (channel A), aged SSA (channel B), and in SMA with aged SSA (channel C) (Figure S4), concentrations of quantified organosulfates in PM_2.5_ nascent (channel A) and aged SSA (channel B), and in SMA with aged SSA (channel C), collected on 5–6 August 2019 (Figure S5), distribution of fatty acids and fatty acid derivatives observed in PM_2.5_ nascent (channel A), aged SSA (channel B), and in SMA with aged SSA (channel C) on 5–6 August 2019 (Figure S6), distribution of alkyl amines observed in PM_1.0_ nascent (channel A), aged SSA (channel B), and in SMA with aged SSA (channel C) on 3–4 August 2019 (peak of the bloom) (Figure S7), concentration of quantified and semi-quantified organosulfates in PM_1.0_ nascent (channel A), aged SSA (channel B) and in SMA with aged SSA (channel C) on sampling days during the peak of the bloom and post-bloom period (Table S1), homologous series of fatty acid derived OS in PM_1.0_ SSA in aged SSA (channel B) (Table S2) (PDF)
