# Supplementary material for: Molecular Characterization of Nascent and Aged Sea Spray Aerosol
Source: ACS Earth Space Chem. 2025 May 19;9(6):1453–64. doi: 10.1021/acsearthspacechem.4c00412 (PMC12183759; doi:10.1021/acsearthspacechem.4c00412)
Supplement: Supplementary file 1 [file sp4c00412_si_001.pdf]

Supporting Information

Molecular characterization of nascent and aged sea spray aerosol

Dilini K. Gamage[1], Elias Hasenecz[1], Glorianne Dorcé[1], Kathryn J. Mayer[2], Jon S. Sauer[2], Christopher Lee[3], Kimberly A. Prather[2,3], Elizabeth A. Stone[1]

[1]Department of Chemistry, University of Iowa, Iowa City, Iowa 52242, United States
[2]Department of Chemistry and Biochemistry, University of California, San Diego, La Jolla, California 92093, United States
[3]Scripps Institution of Oceanography, University of California, San Diego, La Jolla, California 92037, United States

Figure S1: Schematic diagram of sampling nascent SSA (channel A), aged SSA (channel B), and SMA with aged SSA (channel C).[1] Cascade impactors provided separation based on aerodynamic diameter in Channels A and B, whereas total suspended particles (TSP) were collected in Channel C. Backup QFF were collected in each channel.

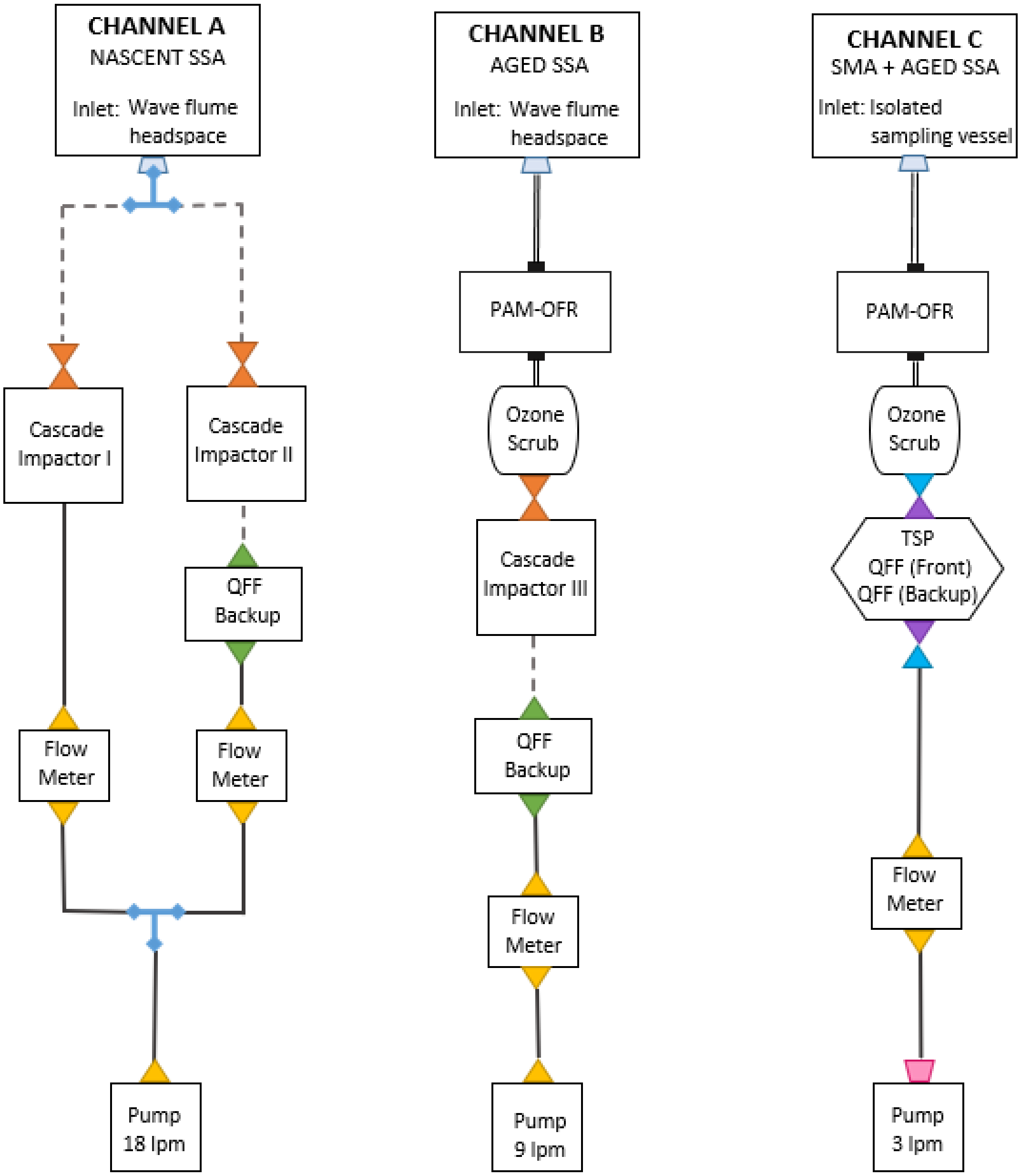

Figure S2: Organic carbon (OC) and inorganic ion concentrations for (a) nascent (channel A) and (b) aged SSA (channel B) on 1-2 August 2019 (peak of the bloom). Other ions include K^+, NH_4^+, F^-, and PO_4^{3-}. The absolute concentrations of inorganic ions measured in channels A and B indicate particle loss in B, particularly for supermicron sized particles. Consequently, intrinsic aerosol properties including ion to Na^+ ratios were used to examine chemical changes resulting from aging.

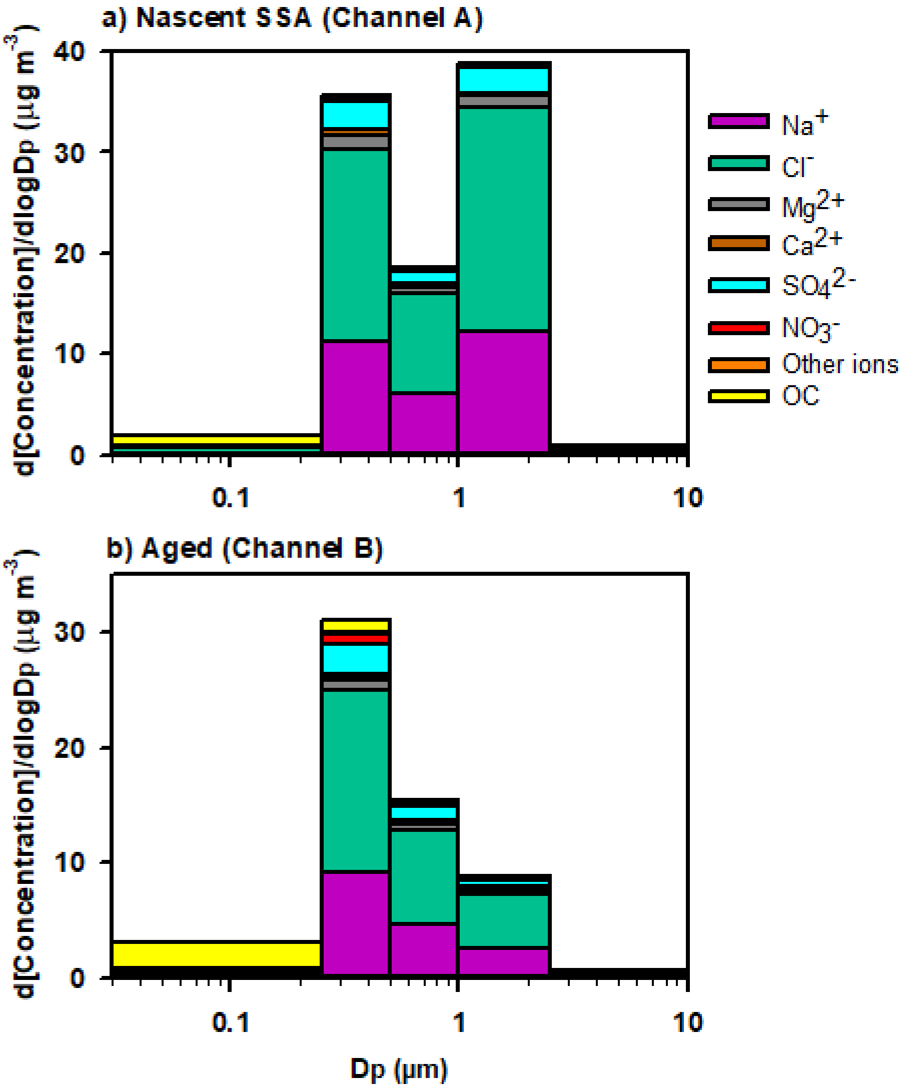

Figure S3: Organic carbon (OC)/sodium(Na^+) ratios for nascent (channel A) and aged SSA (channel B) on (a) 26-27 August (b) 9-10 August 2019 (pre bloom and post bloom). 'BDL' denotes measurements below the detection limit.

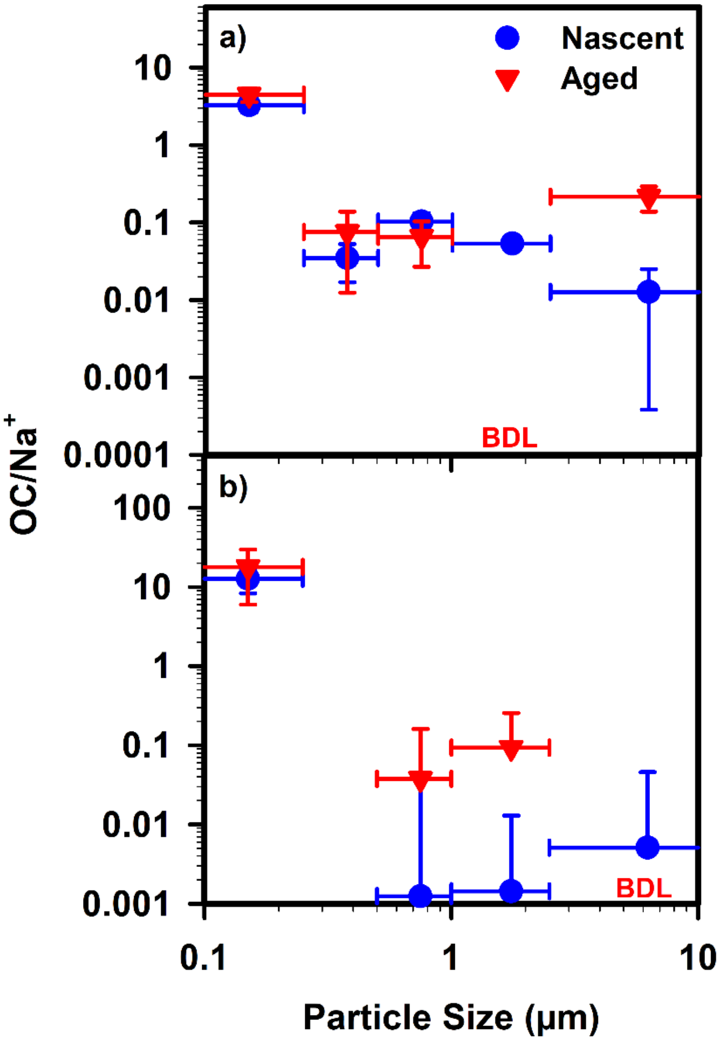

Figure S4: Mass spectra corresponding to precursor ion scans of (A) bisulfate anion (*m/z* 97), and (B) sulfate ion radical (*m/z* 96) in $PM_{1.0}$ nascent (channel A), aged SSA (channel B), and in SMA with aged SSA (channel C).

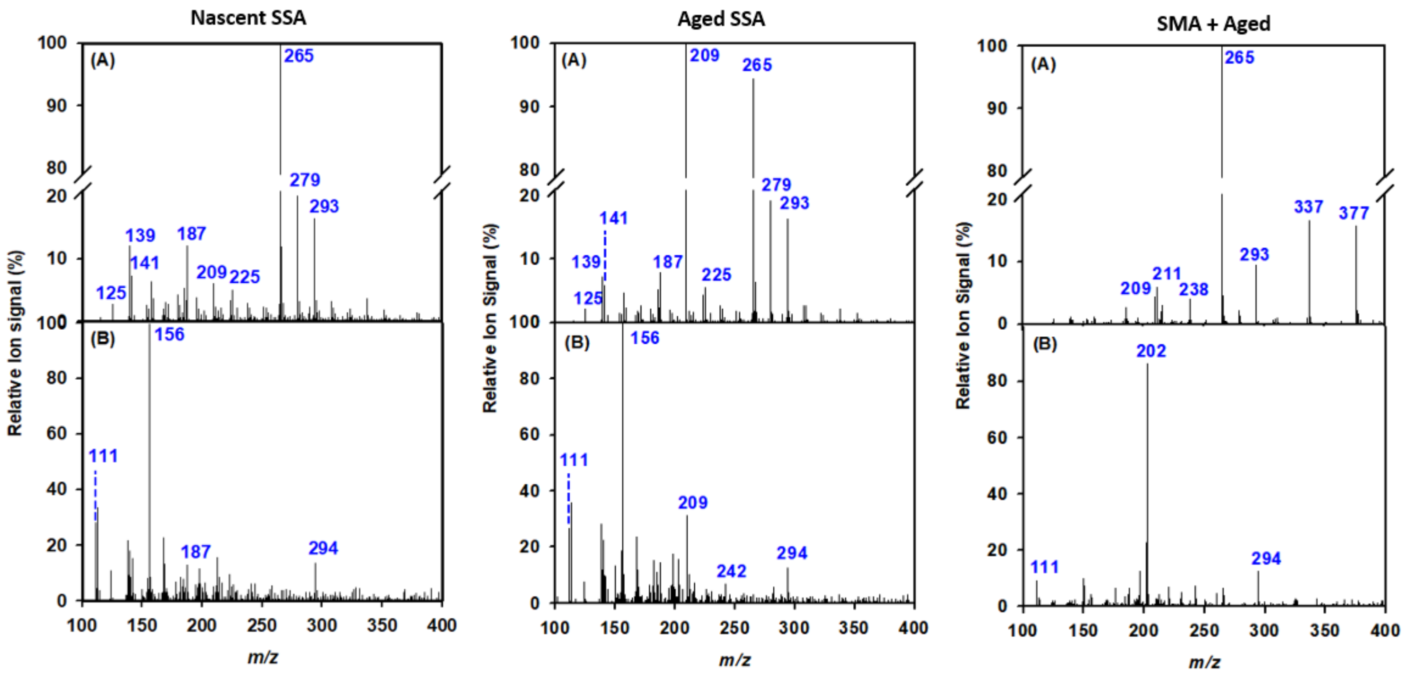

Figure S5: Concentrations of quantified organosulfates in $PM_{2.5}$ nascent (channel A) and $PM_{2.5}$ aged SSA (channel B), and in TSP containing SMA andaged SSA (channel C), collected on 5-6 August 2019. Organosulfates are color-coded purple for low molecular weight species, green for isoprene-derived, and red for monoterpene-derived. Asterisks indicates values below the detection limit (BDL).

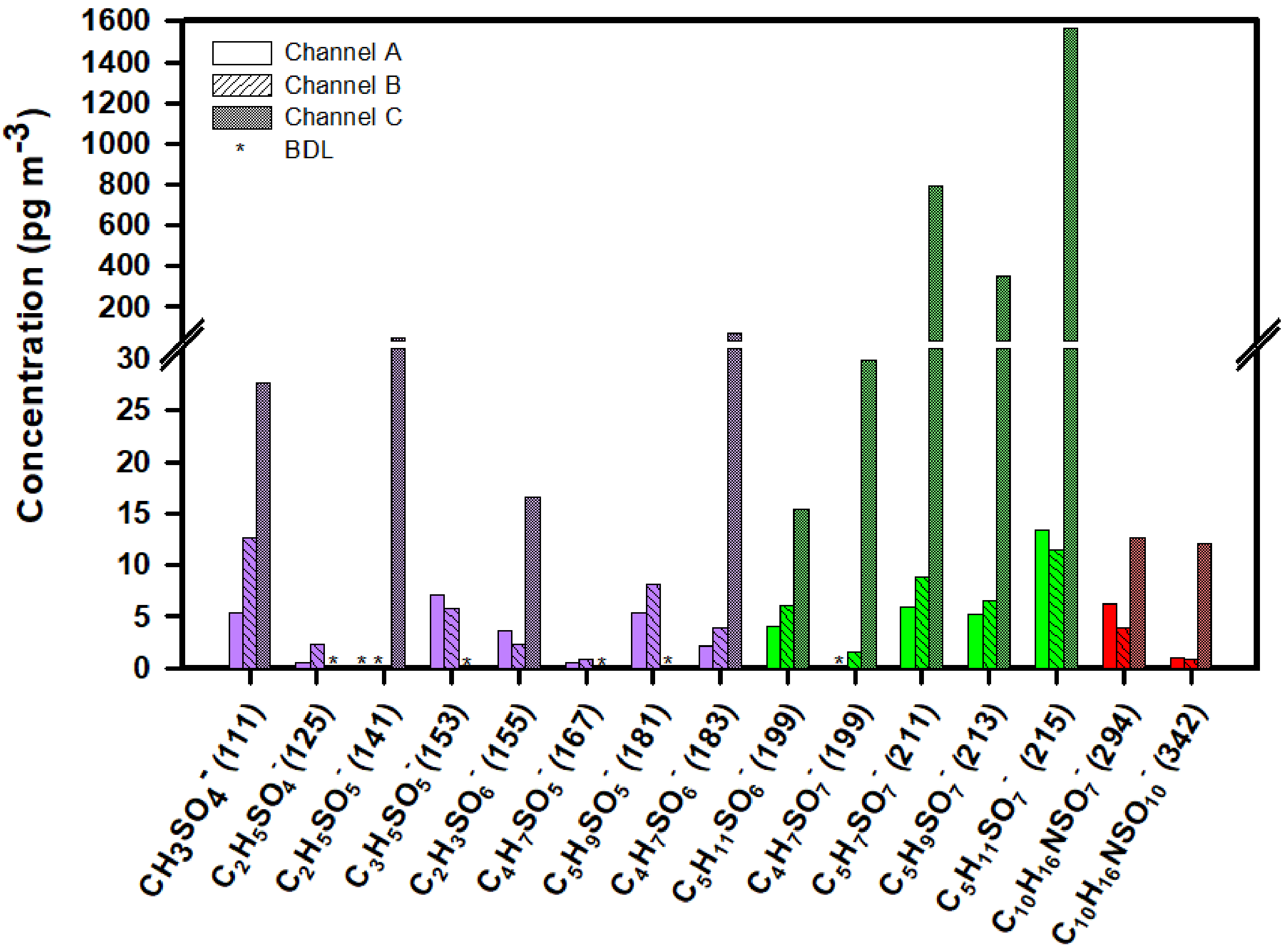

Figure S6: Distribution of fatty acids and fatty acid derivatives observed in $PM_{2.5}$ nascent (channel A), aged SSA (channel B), and in SMA with aged SSA (channel C) on 5-6 August 2019. Responses of each class of compound were obtained from HRMS data from Q-Exactive-Orbitrap-MS, with ESI in negative mode.

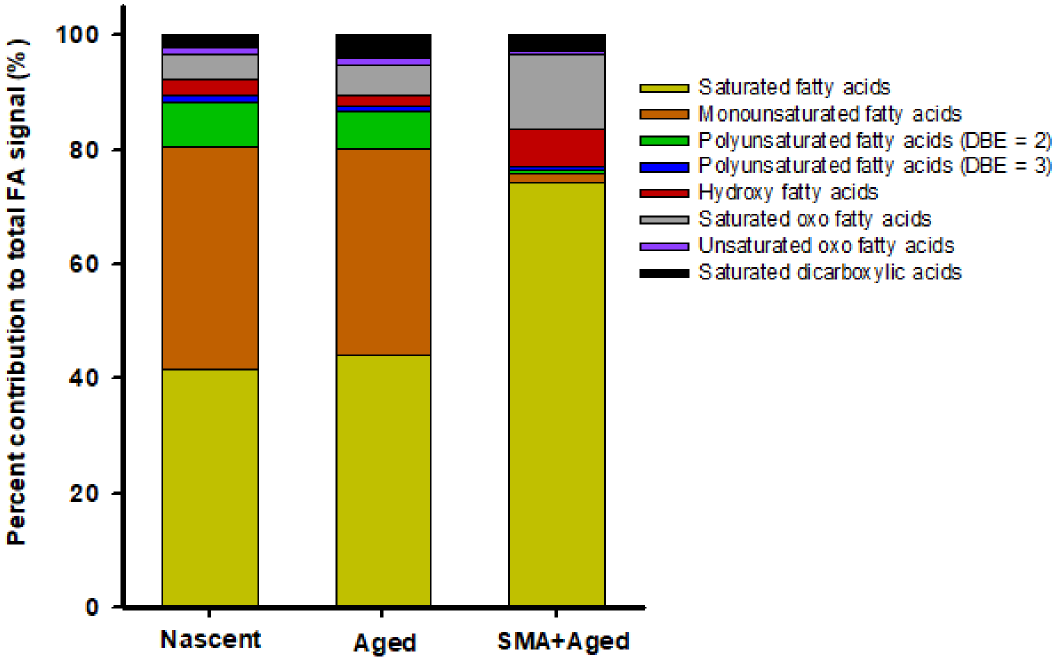

Carbon preference index (CPI) for FA_{sat}

Saturated fatty acids (FA_{sat}) derived from biological sources usually exhibit a tendency to favor compounds that contain an even number of carbon atoms.[2-4] The influence of biological origins on FA_{sat} levels can be emphasized by examining their overall carbon preference index.[5, 6]

$$\mathrm{CPI} = \frac{\sum(\mathrm{C8} - \mathrm{C24})}{\sum(\mathrm{C9} - \mathrm{C25})}$$

CPI values for FA_{sat} in nascent, aged and SMA were 2.5, 2.6 and 9.0 respectively. The higher CPI value observed in channel C compared to nascent and aged SSA could be due to the difference in particle size collected: nascent and aged SSA were $PM_{2.5}$, whereas total suspend particles were collected in channel C. CPI values greater than 1 indicates the biosynthetic pathway of FA_{sat} in organisms, which preferentially produces even-numbered carbon chains, and indicates the influence of biological activity within the wave flume system on FA_{sat} distributions.

Figure S7: Distribution of alkyl amines observed in $PM_{1.0}$ nascent (channel A), aged SSA (channel B), and in SMA with aged SSA (channel C) on 3-4 August 2019 (peak of the bloom). Response of each alkyl amine was obtained from HRMS data from Q-Exactive-Orbitrap-MS, with ESI in positive mode.

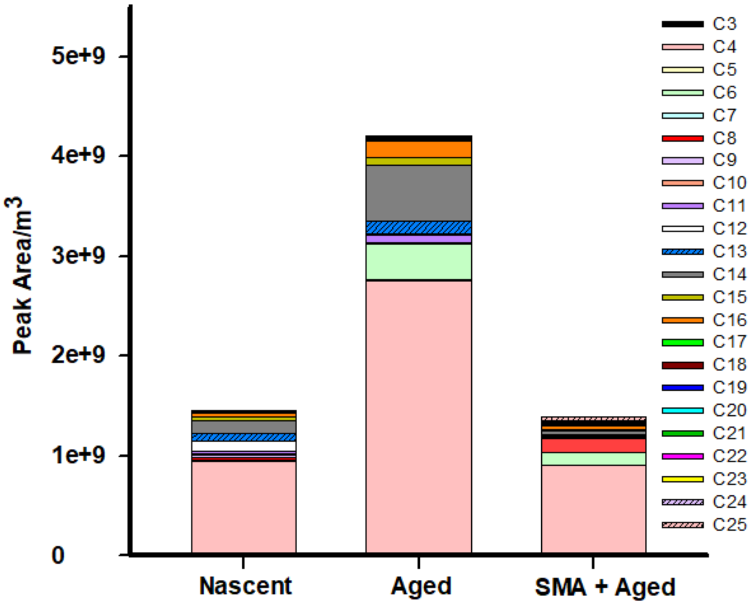

Table S1: Concentration of quantified and semi quantified organosulfates in $PM_{1.0}$ nascent (channel A), aged SSA (channel B) and in SMA with aged SSA (channel C) on sampling days during the peak of the bloom and post-bloom period

Chemical formula $[M–H]^-$	*m/z*	Concentration range (median) (pg m^{-3})			Semi-quantified standard	Retention time (min)
		Nascent SSA	Aged SSA	SMA + Aged SSA		
Methyl sulfate ($CH_3SO_4^-$)	111	ND - 114 (2.4)	BDL - 2.9 (1.5)	34.4 - 80.9 (57.7)	-	0.87
Ethyl sulfate ($C_2H_5SO_4^-$)	125	ND -21.8 (10.9)	BDL - 0.2 (0.1)	BDL -19.8 (9.9)	-	0.76
Benzyl sulfate ($C_7H_7SO_4^-$)	187	ND - 0.1 (0.1)	0.1 - 0.7 (0.4)	1.2 - 19.8 (10.5)	-	0.57
Acetoin sulfate ($C_4H_7SO_5^-$)	167	0.2 - 9.7 (0.4)	2.6 - 3.4 (3.0)	4.8 - 7.6 (6.2)	-	0.60, 0.67, 0.76
Dodecyl sulfate ($C_{12}H_{25}SO_4^-$)	265	BDL - 4240 (2120)	BDL	BDL-161 (80.7)	-	0.52
Hydroxyacetone sulfate ($C_3H_5SO_5^-$)	153	7.2 - 111 (38.6)	11.6 - 16.5 (14.1)	22.1 - 88.5 (55.3)	-	0.68
Glycolic acid sulfate ($C_2H_3SO_6^-$)	155	BDL	5.9 - 8.9 (7.4)	426 - 467 (447)	-	6.94
$C_3H_5SO_4^-$	137	ND	15.5 - 36.1 (25.8)	ND - 27.9 (14.0)	HAS	1.22
$C_2H_5SO_5^-$	141	5.8 - 138 (68.3)	5.9 - 7.9 (6.9)	BDL -104 (51.8)	HAS	1.56
$C_3H_7SO_5^-$	155	0.4 -21.0 (5.6)	11.1 - 15.1 (13.1)	29.0 - 36.6 (32.8)	HAS	1.12
$C_4H_5SO_5^-$	165	ND	0.1	ND - 0.8 (0.4)	HAS	0.57
Lactic acid sulfate ($C_3H_5SO_6^-$)	169	ND - 9.3 (4.6)	5.7 - 11.2 (8.4)	78.1 - 205 (141)	GAS	6.74
$C_5H_9SO_5^-$	181	11.5 - 30.2 (16.2)	21.6 - 21.7 (21.7)	BDL - 56.5 (28.2)	HAS	0.65, 0.89
$C_4H_7SO_6^-$	183	4.3 - 23.9 (11.7)	12.6 - 24.8 (18.7)	77.8 - 92.9 (85.4)	HAS	0.78, 0.95, 1.09
$C_5H_9SO_6^-$	197	6.6 - 38.9 (15.4)	11.5 -20.7 (16.1)	37.7 - 38.2 (37.9)	HAS	0.60, 0.81, 0.98, 1.1, 1.22, 1.55, 1.69, 1.87
$C_5H_{11}SO_6^-$	199	8.3 - 36.4 (18.7)	11.4 - 15.1 (13.2)	29.1 - 42.7 (35.9)	HAS	0.96, 1.54
2-MGA Sulfate ($C_4H_7SO_7^-$)	199	BDL	0.5 - 5.8 (3.2)	52.5 - 238 (145)	GAS	7.21
$C_5H_7SO_7^-$	211	11.0 - 22.9 (18.1)	16.4 - 24.8 (20.6)	511 - 1060 (787)	HAS	0.53, 0.65, 0.81
$C_5H_9SO_7^-$	213	11.5 - 59.5 (34.8)	12.7 - 14.8 (13.8)	258 - 422 (340)	HAS	1.69, 1.80
$C_5H_{11}SO_7^-$	215	14.0 - 27.7 (19.6)	18.2 - 28.5 (23.3)	759 - 1420 (1090)	HAS	1.28, 1.51, 2.31, 2.69, 3.34, 3.77
$C_7H_{11}SO_7^-$	239	14.2 - 38.5 (15.3)	30.0 - 38.0 (34.0)	98.9 - 116 (108)	HAS	0.58, 0.65, 0.72, 0.87, 1.11, 1.18, 1.33, 1.58
$C_{10}H_{16}NSO_7^-$	294	1.0 - 7.5 (4.8)	0.9 - 8.0 (4.4)	72.5 - 119 (95.8)	MS	0.55
$C_{10}H_{16}NSO_{10}^-$	342	1.3 - 22.4 (2.5)	1.7 - 4.5 (3.1)	21.4 - 33.6 (27.5)	HAS	0.51

OS that were quantified are not marked; those that were semi-quantified are noted along with the standard used for the quantification. Methyl sulfate (MS), ethyl sulfate, dodecyl sulfate, acetoin sulfate, benzyl sulfate, hydroxyacetone sulfate (HAS), and glycolic acid sulfate (GAS) were quantified using authentic standards. HAS served as the surrogate for semi-quantifying OS that fragmented to the bisulfate anion (*m/z* 97) and eluted within four minutes. For those eluting after four minutes, GAS was used. MS was the surrogate for OS that fragmented solely to the sulfate radical anion (*m/z* 96). Nascent SSA was collected in channel A, aged SSA in channel B, and Secondary Marine Aerosol (SMA), along with aged SSA was produced in channel C.

Table S2: Homologous series of fatty acid derived OS in $PM_{1.0}$ SSA in aged SSA (channel B). The double bond equivalence (DBE) value for these OS is 1.5.

Formula	Calculated mass (Da)	Error (mDa)	t_R (HRMS) (min)
$C_6H_{11}SO_6^-$	211.0276	– 0.71	0.94
$C_7H_{13}SO_6^-$	225.0433	– 0.58	0.79
$C_8H_{15}SO_6^-$	239.0589	– 0.31	0.51
$C_9H_{17}SO_6^-$	253.0746	0.31	0.6
$C_{10}H_{19}SO_6^-$	267.0902	0.32	0.55
$C_{12}H_{23}SO_6^-$	295.1215	– 0.09	0.74
$C_{13}H_{25}SO_6^-$	309.1372	– 0.41	0.55, 0.70
$C_{14}H_{27}SO_6^-$	323.1528	– 0.65	0.52-0.79
$C_{15}H_{29}SO_6^-$	337.1685	0.53	0.58, 1.13
$C_{16}H_{31}SO_6^-$	351.1841	– 0.63	0.52, 0.57, 1.86
$C_{18}H_{35}SO_6^-$	379.2154	– 0.51	0.52, 1.53
$C_{19}H_{37}SO_6^-$	393.2311	– 0.58	0.51

References

(1) Sauer, J. S.; Mayer, K. J.; Mayer, K. J.; Lee, C.; Alves, M. R.; Amiri, S.; Bahaveolos, C. J.; Franklin, E. B.; Crocker, D. R.; Dang, D.; Dinasquet, J.; Garofalo, L. A.; Kaluarachchi, C. P.; Kilgour, D. B.; Mael, L. E.; Mitts, B. A.; Moon, D. R.; Moore, A. N.; Morris, C. K.; Mullenmeister, C. A.; Ni, C.-M.; Pendergraft, M. A.; Petras, D.; Simpson, R. M. C.; Smith, S.; Tumminello, P. R.; Walker, J. L.; DeMott, P. J.; Farmer, D. K.; Goldstein, A. H.; Grassian, V. H.; Jaffe, J. S.; Malfatti, F.; Martz, T. R.; Slade, J. H.; Tivanski, A. V.; Bertram, T. H.; Cappa, C. D.; Prather, K. A. The Sea Spray Chemistry and Particle Evolution study (SeaSCAPE): overview and experimental methods. Environ. Sci. Proc. Imp. 2022, 24 (2), 290-315. DOI: 10.1039/d1em00260k.

(2) Roach, P. J.; Laskin, J.; Laskin, A. Higher-Order Mass Defect Analysis for Mass Spectra of Complex Organic Mixtures. Anal. Chem. 2011, 83 (12), 4924-4929. DOI: 10.1021/ac200654j.

(3) Mochida, M.; Kitamori, Y.; Kawamura, K.; Nojiri, Y.; Suzuki, K. Fatty acids in the marine atmosphere: Factors governing their concentrations and evaluation of organic films on sea-salt particles. J. Geophys. 2002, 107 (D17), AAC 1-1-AAC 1-10. DOI: 10.1029/2001JD001278.

(4) Zhang, Y. X.; Shao, M.; Zhang, Y. H.; Zeng, L. M.; He, L. Y.; Zhu, B.; Wei, Y. J.; Zhu, X. L. Source profiles of particulate organic matters emitted from cereal straw burnings. J. Environ. Sci-China 2007, 19 (2), 167-175. DOI: 10.1016/S1001-0742(07)60027-8.

(5) Tyagi, P.; Ishimura, Y.; Kawamura, K. Hydroxy fatty acids in marine aerosols as microbial tracers: 4-year study on β- and ω-hydroxy fatty acids from remote Chichijima Island in the western North Pacific. Atmos. Environ. 2015, 115, 89-100. DOI: 10.1016/j.atmosenv.2015.05.038.

(6) Roslan, R. N.; Hanif, N. M.; Othman, M. R.; Azmi, W. N. F. W.; Yan, X. X.; Ali, M. M.; Mohamed, C. A. R.; Latif, M. T. Surfactants in the sea-surface microlayer and their contribution to atmospheric aerosols around coastal areas of the Malaysian peninsula. Mar. Pollut. Bull. 2010, 60 (9), 1584-1590. DOI: 10.1016/j.marpolbul.2010.04.004.
